# *N*-(4-Bromo­phen­yl)-3,4,5-trimeth­oxy­benzamide

**DOI:** 10.1107/S1600536812018946

**Published:** 2012-05-05

**Authors:** Wen Gu, Chao Qiao

**Affiliations:** a College of Chemical Engineering, Nanjing Forestry University, Nanjing 210037, People’s Republic of China

## Abstract

In the title compound, C_16_H_16_BrNO_4_, the dihedral angle between the two aromatic rings is 67.51 (25)°. In the crystal, mol­ecules are linked by N—H⋯O hydrogen bonds involving the N—H and C=O groups of the amide function, leading to a chain along [-101].

## Related literature

For the synthesis and biological activity of 3,4,5-trimeth­oxy­benzamide derivatives, see: Buettner *et al.* (2009 ▶); Pellicani *et al.* (2012 ▶). For related structures, see: Saeed & Flörke (2009 ▶); Saeed *et al.* (2008 ▶); Choi *et al.* (2010 ▶).
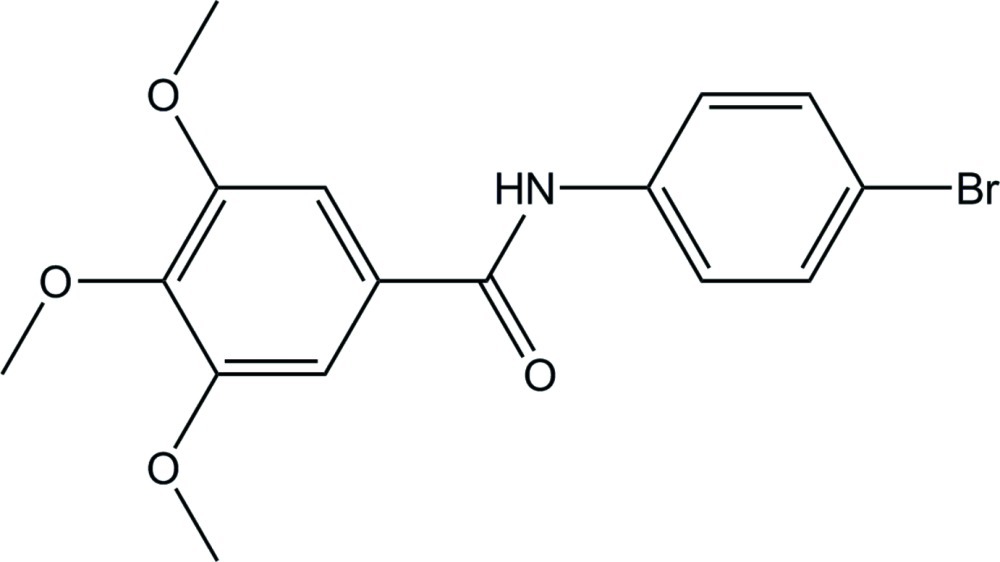


## Experimental

### 

#### Crystal data

C_16_H_16_BrNO_4_
*M* *_r_* = 366.21
Monoclinic, 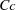

*a* = 9.5860 (19) Å
*b* = 26.010 (5) Å
*c* = 7.1390 (14) Å
β = 112.04 (3)°
*V* = 1649.9 (6) Å^3^
*Z* = 4
Mo *K*α radiation
μ = 2.51 mm^−1^
*T* = 293 K
0.20 × 0.10 × 0.10 mm

#### Data collection

Enraf–Nonius CAD-4 diffractometer
Absorption correction: ψ scan (North *et al.*, 1968 ▶) *T*_min_ = 0.634, *T*_max_ = 0.788
3194 measured reflections
1616 independent reflections
1206 reflections with *I* > 2σ(*I*)
*R*_int_ = 0.064
3 standard reflections every 200 reflections intensity decay: 1%

#### Refinement

*R*[*F* ^2^ > 2σ(*F* ^2^)] = 0.045
*wR*(*F* ^2^) = 0.094
*S* = 1.00
1616 reflections
199 parameters
2 restraints
H-atom parameters constrained
Δρ_max_ = 0.37 e Å^−3^
Δρ_min_ = −0.26 e Å^−3^
Absolute structure: Flack (1983 ▶), 91 Friedel pairs
Flack parameter: 0.010 (17)

### 

Data collection: *CAD-4 EXPRESS* (Enraf–Nonius, 1989 ▶); cell refinement: *CAD-4 EXPRESS*; data reduction: *XCAD4* (Harms & Wocadlo, 1995 ▶); program(s) used to solve structure: *SHELXS97* (Sheldrick, 2008 ▶); program(s) used to refine structure: *SHELXL97* (Sheldrick, 2008 ▶); molecular graphics: *SHELXTL* (Sheldrick, 2008 ▶); software used to prepare material for publication: *SHELXL97*.

## Supplementary Material

Crystal structure: contains datablock(s) I, global. DOI: 10.1107/S1600536812018946/kp2410sup1.cif

Structure factors: contains datablock(s) I. DOI: 10.1107/S1600536812018946/kp2410Isup2.hkl

Supplementary material file. DOI: 10.1107/S1600536812018946/kp2410Isup3.cml

Additional supplementary materials:  crystallographic information; 3D view; checkCIF report

## Figures and Tables

**Table 1:** Hydrogen-bond geometry (Å, °)

*D*—H⋯*A*	*D*—H	H⋯*A*	*D*⋯*A*	*D*—H⋯*A*
N—H0*A*⋯O4^i^	0.86	2.19	2.909 (9)	140

Symmetry code: (i) 
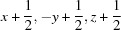
.

**Fig. 1.**
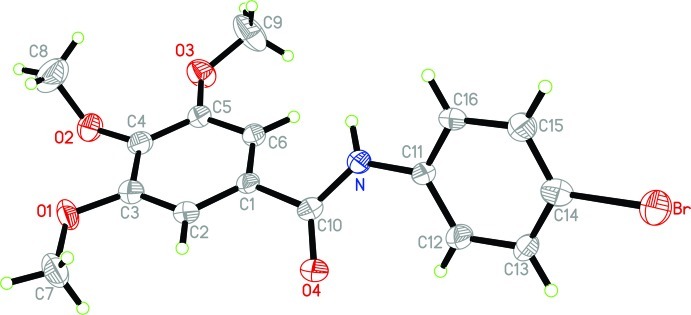
Molecular structure of (I) with 30% probability displacement ellipsoids for non-H atoms.

## supplementary crystallographic information

### Comment

As a part of our ongoing research on the synthesis and biological activities of 3,4,5-trimethoxy-benzamide derivatives, the title compound (I) was synthesised and its crystal structure was determined (Fig. 1). In the crystal packing N-H···O hydrogen bond generates a chain along [101] (Table 1).

### Experimental

To a solution of 3,4,5-Trimethoxybenzoyl chloride (1.15 g, 5 mmol) in benzene (20 mL) was added 4-bromoaniline (0.95 g, 5.5 mmol) and triethylamine (0.56 g, 5.5 mmol). The mixture was stirred at room temperature for 12 h. After cooling, the reaction mixture was filtered to remove precipitate, and the filtrate was evaporated *in vacuo* to afford a white solid, which was recrystalised in EtOH to give the title compound (I) as a colourless prisms (1.5 g, 82%). Single crystals of (I) suitable for X-ray diffraction study were obtained by slow evaporation of an ethanol solution at room temperature over 7 d.

### Refinement

All H atoms were placed in idealized positions with C—H = 0.93 or 0.96 Å, N—H = 0.86 Å, and refined using a riding model with *U*_iso_(H) = 1.2*U*_eq_ (C,*N*), or 1.5*U*_eq_ for methyl-C.

### Crystal data

**Table:**

C_16_H_16_BrNO_4_	*F*(000) = 744
*M**_r_* = 366.21	*D*_x_ = 1.474 Mg m^−^^3^
Monoclinic, *C**c*	Mo *K*α radiation, λ = 0.71073 Å
Hall symbol: C -2yc	Cell parameters from 25 reflections
*a* = 9.5860 (19) Å	θ = 10–13°
*b* = 26.010 (5) Å	µ = 2.51 mm^−^^1^
*c* = 7.1390 (14) Å	*T* = 293 K
β = 112.04 (3)°	Block, colourless
*V* = 1649.9 (6) Å^3^	0.20 × 0.10 × 0.10 mm
*Z* = 4	

### Data collection

**Table:**

Enraf–Nonius CAD-4 diffractometer	1206 reflections with *I* > 2σ(*I*)
Radiation source: fine-focus sealed tube	*R*_int_ = 0.064
Graphite monochromator	θ_max_ = 25.4°, θ_min_ = 1.6°
ω/2θ scans	*h* = 0→11
Absorption correction: ψ scan (North *et al.*, 1968)	*k* = −31→31
*T*_min_ = 0.634, *T*_max_ = 0.788	*l* = −8→7
3194 measured reflections	3 standard reflections every 200 reflections
1616 independent reflections	intensity decay: 1%

### Refinement

**Table:**

Refinement on *F*^2^	Secondary atom site location: difference Fourier map
Least-squares matrix: full	Hydrogen site location: inferred from neighbouring sites
*R*[*F*^2^ > 2σ(*F*^2^)] = 0.045	H-atom parameters constrained
*wR*(*F*^2^) = 0.094	*w* = 1/[σ^2^(*F*_o_^2^) + (0.043*P*)^2^] where *P* = (*F*_o_^2^ + 2*F*_c_^2^)/3
*S* = 1.00	(Δ/σ)_max_ < 0.001
1616 reflections	Δρ_max_ = 0.37 e Å^−^^3^
199 parameters	Δρ_min_ = −0.26 e Å^−^^3^
2 restraints	Absolute structure: Flack (1983), 91 Friedel pairs
Primary atom site location: structure-invariant direct methods	Flack parameter: 0.010 (17)

### Special details

**Table:**

Geometry. All e.s.d.'s (except the e.s.d. in the dihedral angle between two l.s. planes) are estimated using the full covariance matrix. The cell e.s.d.'s are taken into account individually in the estimation of e.s.d.'s in distances, angles and torsion angles; correlations between e.s.d.'s in cell parameters are only used when they are defined by crystal symmetry. An approximate (isotropic) treatment of cell e.s.d.'s is used for estimating e.s.d.'s involving l.s. planes.
Refinement. Refinement of *F*^2^ against ALL reflections. The weighted *R*-factor *wR* and goodness of fit *S* are based on *F*^2^, conventional *R*-factors *R* are based on *F*, with *F* set to zero for negative *F*^2^. The threshold expression of *F*^2^ > σ(*F*^2^) is used only for calculating *R*-factors(gt) *etc*. and is not relevant to the choice of reflections for refinement. *R*-factors based on *F*^2^ are statistically about twice as large as those based on *F*, and *R*- factors based on ALL data will be even larger.

### Fractional atomic coordinates and isotropic or equivalent isotropic displacement parameters (Å^2^)

**Table:**

	*x*	*y*	*z*	*U*_iso_*/*U*_eq_	
Br	0.49808 (11)	0.47594 (3)	0.04427 (12)	0.0691 (3)	
N	0.5036 (10)	0.28535 (15)	0.5367 (13)	0.0464 (12)	
H0A	0.5729	0.2840	0.6555	0.056*	
O1	0.4265 (6)	0.05888 (17)	0.6046 (7)	0.0626 (14)	
C6	0.5016 (7)	0.2076 (3)	0.8251 (10)	0.0440 (17)	
H6A	0.5203	0.2407	0.8776	0.053*	
C5	0.5285 (8)	0.1651 (3)	0.9537 (11)	0.0429 (17)	
O2	0.5191 (7)	0.07461 (17)	0.9997 (8)	0.0588 (15)	
O3	0.5812 (7)	0.1688 (2)	1.1618 (7)	0.0637 (15)	
C4	0.5010 (8)	0.1162 (3)	0.8755 (10)	0.0479 (18)	
O4	0.3061 (5)	0.24199 (18)	0.3087 (8)	0.0559 (13)	
C3	0.4450 (8)	0.1084 (3)	0.6641 (11)	0.0485 (18)	
C2	0.4170 (8)	0.1506 (3)	0.5388 (11)	0.0473 (17)	
H2A	0.3778	0.1461	0.3994	0.057*	
C1	0.4467 (7)	0.1996 (2)	0.6193 (10)	0.0364 (15)	
C7	0.3625 (12)	0.0497 (3)	0.3925 (12)	0.077 (3)	
H7A	0.3546	0.0134	0.3677	0.115*	
H7B	0.4256	0.0647	0.3297	0.115*	
H7C	0.2642	0.0650	0.3372	0.115*	
C8	0.6627 (12)	0.0518 (4)	1.0685 (17)	0.102 (3)	
H8A	0.6647	0.0231	1.1541	0.153*	
H8B	0.7367	0.0766	1.1435	0.153*	
H8C	0.6846	0.0402	0.9549	0.153*	
C9	0.6542 (13)	0.2131 (3)	1.2509 (12)	0.090 (3)	
H9A	0.6845	0.2104	1.3949	0.134*	
H9B	0.5876	0.2418	1.2027	0.134*	
H9C	0.7414	0.2178	1.2174	0.134*	
C10	0.4106 (8)	0.2446 (3)	0.4759 (11)	0.0456 (17)	
C11	0.4968 (7)	0.3301 (2)	0.4216 (10)	0.0403 (15)	
C12	0.3606 (8)	0.3507 (3)	0.2917 (11)	0.0508 (18)	
H12A	0.2701	0.3352	0.2792	0.061*	
C13	0.3614 (9)	0.3948 (2)	0.1802 (11)	0.054 (2)	
H13A	0.2714	0.4088	0.0924	0.064*	
C14	0.4971 (8)	0.4173 (3)	0.2018 (10)	0.0514 (19)	
C15	0.6283 (9)	0.3986 (3)	0.3347 (12)	0.057 (2)	
H15A	0.7182	0.4154	0.3539	0.068*	
C16	0.6286 (8)	0.3538 (3)	0.4433 (11)	0.0515 (19)	
H16B	0.7193	0.3402	0.5308	0.062*	

### Atomic displacement parameters (Å^2^)

**Table:**

	*U*^11^	*U*^22^	*U*^33^	*U*^12^	*U*^13^	*U*^23^
Br	0.0741 (5)	0.0650 (5)	0.0620 (4)	−0.0090 (6)	0.0184 (4)	0.0183 (5)
N	0.047 (3)	0.038 (3)	0.041 (3)	−0.004 (4)	0.002 (2)	0.008 (4)
O1	0.084 (4)	0.029 (3)	0.061 (3)	−0.007 (3)	0.011 (3)	−0.003 (2)
C6	0.032 (4)	0.041 (4)	0.057 (5)	0.002 (3)	0.014 (3)	−0.002 (3)
C5	0.038 (4)	0.043 (4)	0.045 (4)	0.002 (3)	0.012 (3)	0.002 (3)
O2	0.065 (4)	0.048 (3)	0.057 (4)	0.001 (3)	0.016 (3)	0.016 (3)
O3	0.081 (4)	0.064 (4)	0.040 (3)	−0.013 (3)	0.016 (3)	−0.002 (2)
C4	0.039 (4)	0.050 (5)	0.050 (4)	0.000 (3)	0.011 (3)	0.008 (3)
O4	0.041 (3)	0.051 (3)	0.059 (3)	−0.006 (3)	0.000 (3)	0.001 (2)
C3	0.043 (4)	0.047 (4)	0.051 (5)	−0.001 (3)	0.012 (4)	0.001 (3)
C2	0.040 (4)	0.046 (4)	0.045 (4)	−0.001 (3)	0.004 (3)	0.001 (3)
C1	0.027 (3)	0.038 (4)	0.041 (4)	0.002 (3)	0.010 (3)	0.003 (3)
C7	0.107 (7)	0.044 (5)	0.065 (5)	−0.008 (5)	0.016 (5)	−0.014 (4)
C8	0.089 (8)	0.086 (7)	0.109 (8)	0.022 (6)	0.013 (7)	0.042 (6)
C9	0.143 (10)	0.080 (6)	0.047 (5)	−0.047 (6)	0.037 (6)	−0.025 (4)
C10	0.034 (4)	0.046 (4)	0.050 (5)	0.005 (3)	0.008 (4)	0.002 (3)
C11	0.035 (4)	0.037 (4)	0.042 (4)	0.003 (3)	0.006 (3)	−0.001 (3)
C12	0.036 (4)	0.049 (4)	0.060 (4)	0.001 (3)	0.009 (4)	0.009 (3)
C13	0.042 (4)	0.045 (4)	0.062 (5)	0.004 (4)	0.006 (4)	0.018 (4)
C14	0.047 (5)	0.060 (5)	0.045 (4)	−0.006 (4)	0.015 (4)	−0.006 (3)
C15	0.046 (5)	0.054 (5)	0.064 (5)	−0.006 (4)	0.014 (4)	0.012 (4)
C16	0.039 (4)	0.048 (4)	0.051 (4)	−0.008 (3)	−0.003 (3)	0.003 (3)

### Geometric parameters (Å, º)

**Table:**

Br—C14	1.898 (7)	C7—H7A	0.9600
N—C10	1.347 (9)	C7—H7B	0.9600
N—C11	1.413 (8)	C7—H7C	0.9600
N—H0A	0.8600	C8—H8A	0.9600
O1—C3	1.347 (8)	C8—H8B	0.9600
O1—C7	1.424 (9)	C8—H8C	0.9600
C6—C1	1.378 (9)	C9—H9A	0.9600
C6—C5	1.396 (10)	C9—H9B	0.9600
C6—H6A	0.9300	C9—H9C	0.9600
C5—C4	1.375 (10)	C11—C16	1.361 (9)
C5—O3	1.382 (8)	C11—C12	1.394 (9)
O2—C4	1.368 (8)	C12—C13	1.397 (9)
O2—C8	1.407 (11)	C12—H12A	0.9300
O3—C9	1.372 (9)	C13—C14	1.381 (10)
C4—C3	1.414 (10)	C13—H13A	0.9300
O4—C10	1.239 (8)	C14—C15	1.350 (10)
C3—C2	1.377 (9)	C15—C16	1.398 (10)
C2—C1	1.383 (9)	C15—H15A	0.9300
C2—H2A	0.9300	C16—H16B	0.9300
C1—C10	1.506 (9)		
			
C10—N—C11	125.5 (8)	H8A—C8—H8B	109.5
C10—N—H0A	117.2	O2—C8—H8C	109.5
C11—N—H0A	117.2	H8A—C8—H8C	109.5
C3—O1—C7	116.6 (6)	H8B—C8—H8C	109.5
C1—C6—C5	119.0 (7)	O3—C9—H9A	109.5
C1—C6—H6A	120.5	O3—C9—H9B	109.5
C5—C6—H6A	120.5	H9A—C9—H9B	109.5
C4—C5—O3	116.0 (7)	O3—C9—H9C	109.5
C4—C5—C6	120.3 (7)	H9A—C9—H9C	109.5
O3—C5—C6	123.7 (7)	H9B—C9—H9C	109.5
C4—O2—C8	115.4 (6)	O4—C10—N	123.4 (7)
C9—O3—C5	118.3 (6)	O4—C10—C1	120.6 (6)
O2—C4—C5	120.7 (6)	N—C10—C1	115.9 (6)
O2—C4—C3	118.9 (6)	C16—C11—C12	120.0 (6)
C5—C4—C3	120.3 (6)	C16—C11—N	118.0 (6)
O1—C3—C2	125.9 (7)	C12—C11—N	122.0 (7)
O1—C3—C4	115.2 (6)	C11—C12—C13	119.3 (7)
C2—C3—C4	118.8 (7)	C11—C12—H12A	120.3
C3—C2—C1	120.3 (6)	C13—C12—H12A	120.3
C3—C2—H2A	119.8	C14—C13—C12	119.4 (7)
C1—C2—H2A	119.8	C14—C13—H13A	120.3
C6—C1—C2	121.2 (6)	C12—C13—H13A	120.3
C6—C1—C10	120.4 (6)	C15—C14—C13	121.1 (7)
C2—C1—C10	118.3 (6)	C15—C14—Br	119.7 (6)
O1—C7—H7A	109.5	C13—C14—Br	119.3 (6)
O1—C7—H7B	109.5	C14—C15—C16	119.7 (7)
H7A—C7—H7B	109.5	C14—C15—H15A	120.1
O1—C7—H7C	109.5	C16—C15—H15A	120.1
H7A—C7—H7C	109.5	C11—C16—C15	120.4 (7)
H7B—C7—H7C	109.5	C11—C16—H16B	119.8
O2—C8—H8A	109.5	C15—C16—H16B	119.8
O2—C8—H8B	109.5		
			
C1—C6—C5—C4	0.2 (10)	C3—C2—C1—C6	−1.5 (10)
C1—C6—C5—O3	−179.0 (7)	C3—C2—C1—C10	−178.6 (6)
C4—C5—O3—C9	159.9 (8)	C11—N—C10—O4	−0.3 (13)
C6—C5—O3—C9	−20.9 (11)	C11—N—C10—C1	175.8 (7)
C8—O2—C4—C5	−91.1 (9)	C6—C1—C10—O4	−147.3 (7)
C8—O2—C4—C3	92.5 (9)	C2—C1—C10—O4	29.8 (9)
O3—C5—C4—O2	2.9 (10)	C6—C1—C10—N	36.4 (9)
C6—C5—C4—O2	−176.4 (6)	C2—C1—C10—N	−146.4 (7)
O3—C5—C4—C3	179.2 (7)	C10—N—C11—C16	−145.7 (8)
C6—C5—C4—C3	0.0 (10)	C10—N—C11—C12	35.4 (12)
C7—O1—C3—C2	−4.5 (11)	C16—C11—C12—C13	1.8 (10)
C7—O1—C3—C4	176.6 (7)	N—C11—C12—C13	−179.3 (7)
O2—C4—C3—O1	−5.5 (9)	C11—C12—C13—C14	−0.3 (11)
C5—C4—C3—O1	178.1 (6)	C12—C13—C14—C15	−2.7 (11)
O2—C4—C3—C2	175.5 (6)	C12—C13—C14—Br	178.1 (5)
C5—C4—C3—C2	−0.9 (11)	C13—C14—C15—C16	4.2 (12)
O1—C3—C2—C1	−177.2 (7)	Br—C14—C15—C16	−176.7 (6)
C4—C3—C2—C1	1.6 (10)	C12—C11—C16—C15	−0.4 (11)
C5—C6—C1—C2	0.6 (10)	N—C11—C16—C15	−179.3 (8)
C5—C6—C1—C10	177.6 (6)	C14—C15—C16—C11	−2.6 (12)

### Hydrogen-bond geometry (Å, º)

**Table:**

*D*—H···*A*	*D*—H	H···*A*	*D*···*A*	*D*—H···*A*
N—H0*A*···O4^i^	0.86	2.19	2.909 (9)	140

Symmetry code: (i) *x*+1/2, −*y*+1/2, *z*+1/2.
